# Antiviral efficacy of silicon nitride against SARS-CoV-2 and MERS-CoV: implications for PPE innovation

**DOI:** 10.3389/fmicb.2025.1637848

**Published:** 2025-08-25

**Authors:** Brittany N. Heath, Caitlin M. Woodson, Chelsey McMinn, Ryan M. Bock, Kylene Kehn-Hall

**Affiliations:** ^1^Department of Biomedical Sciences and Pathobiology, Virginia-Maryland College of Veterinary Medicine, Virginia Polytechnic Institute and State University, Blacksburg, VA, United States; ^2^Center for Emerging, Zoonotic, and Arthropod-borne Pathogens, Virginia Polytechnic Institute and State University, Blacksburg, VA, United States; ^3^SINTX Technologies, Inc., Salt Lake City, UT, United States

**Keywords:** SARS-CoV-2, MERS-CoV, silicon nitride, Si3N4, antiviral

## Abstract

Medical interventions, such as masks, were a cornerstone in mitigating the spread of severe acute respiratory syndrome coronavirus 2 (SARS-CoV-2). Since 2019, the scientific community has increasingly focused on exploring avenues for pandemic prevention and preparedness to enhance responses to future viral outbreaks. One such area of interest explores the use of additives, such as silicon nitride (Si₃N₄), in masks to enhance the antiviral properties of personal protective equipment. Si₃N₄ powder has been shown to inactivate SARS-CoV-2 under aqueous conditions, with a similar yet modest reduction in virus when embedded into non-woven fabric. This study aimed to optimize the use of Si₃N₄ as an antiviral agent. We addressed this knowledge gap by comparing the viral inactivation potential of β-Si₃N₄ and α-Si₃N₄ powders against multiple SARS-CoV-2 lineages and Middle Eastern respiratory syndrome coronavirus (MERS-CoV). Additionally, we tested a refined non-woven fabric embedded with α-Si₃N₄ against SARS-CoV-2 (Lineage A). The results presented here suggest that α-Si₃N₄ was the most effective in reducing the infectious virus through viral RNA degradation, as determined by plaque assay and RT-qPCR. The most promising finding was the significant reduction of SARS-CoV-2 after only 10 min of exposure to refined α-Si₃N₄-embedded fabric. Thus, silicon nitride could be an invaluable inorganic additive in personal protective equipment during future viral epidemics and pandemics.

## 1 Introduction

Pandemic preparedness has become a central focus in response to the coronavirus disease of 2019 (COVID-19) pandemic and the rising trend in zoonotic RNA viral outbreaks (Meadows et al., 2023; Williams et al., 2023; He et al., 2023). Transmission of severe acute respiratory syndrome coronavirus 2 (SARS-CoV-2) has been challenging to mitigate due to the virus’s particulate size and the ease with which it aerosolizes, allowing aerosols to remain airborne for several hours (Van Doremalen et al., 2020). Additionally, there were increased COVID-19 hospital-acquired infections prior to the strict implementation of non-pharmaceutical interventions, such as spatial separation of three to six feet between individuals, increased frequency of surface sanitization, and adequate ventilation (Bahl et al., 2022; Ngandu et al., 2022). Non-pharmaceutical product development is an area of research and development (R&D) that requires improvement, with a specific need for additional broad-spectrum virucidal interventions that can be implemented before future viral outbreaks (Keusch et al., 2022).

One potential avenue for virucidal intervention is the use of silicon nitride (Si_3_N_4_). Si_3_N_4_ is a non-oxide ceramic that is used in Food and Drug Administration (FDA)-cleared implantable spinal fusion devices, with clinical data showing excellent long-term outcomes in both lumbar and cervical fusion with Si_3_N_4_ compared to other spine biomaterials (Smith et al., 2018; Ball et al., 2017; Arts et al., 2017; Calvert et al., 2019; Calvert et al., 2020; McEntire et al., 2020). In aqueous environments, Si_3_N_4_ implants undergo surface hydrolysis, resulting in microscopic elution of ammonium, which is converted into ammonia, nitric oxide, and other reactive nitrogen species that inhibit bacterial growth and proliferation (Pezzotti, 2019). Silicon nitride exists in two main crystal configurations: α-Si_3_N_4_ and β-Si_3_N_4_, which adopt trigonal and hexagonal structures, respectively. Both structures belong to the hexagonal crystal family and consist of differently arranged layers of corner-sharing SiN_4_ tetrahedra (Wang et al., 1996; Hardie and Jack, 1957). The manufacturing process of Si_3_N_4_ can change the phase of silicon nitride from α phase to β phase (Heimann, 2023). The β-Si_3_N_4_ phase is often used for load-bearing ceramics due to its increased stability and toughness (Zhu and Sakka, 2008).

Silicon nitride has also been shown to exhibit antiviral activity against multiple pathogens, in which an aqueous solution of Si_3_N_4_ particles inactivated influenza A virus H1N1 (Influenza A/Puerto Rico/8/1934), enterovirus (EV-A71), and feline calicivirus (Pezzotti et al., 2021). Further studies revealed that aqueous suspension of Si_3_N_4_ inactivated SARS-CoV-2 Delta (Lineage B.1.617.2) and Kappa (Lineage B.1.617.1) variants, and embedment of β-Si_3_N_4_ and α-Si_3_N_4_ in non-woven fabric led to the reduction of SARS-CoV-2 Alpha (Lineage A) (Pezzotti et al., 2022; Simpson et al., 2023). Therefore, the virucidal activity of silicon nitride should be further explored as a potential early medical intervention in preparation for future epidemics and pandemics.

This research study builds upon existing studies comparing the viral inactivation capabilities of β-Si_3_N_4_ and α-Si_3_N_4_ in aqueous suspension against several SARS-CoV-2 lineages and the Middle Eastern respiratory syndrome coronavirus (MERS-CoV). In addition to expanding the testing of powders for MERS-CoV, we refined the non-woven fabric embedded with α-Si_3_N_4_ and tested the improved fabrics against SARS-CoV-2 (Lineage A).

## 2 Results

### 2.1 Characterization of α-Si_3_N_4_ (AP^4^) and β-Si_3_N_4_ (AP^2^) powders

**Figure 1:**
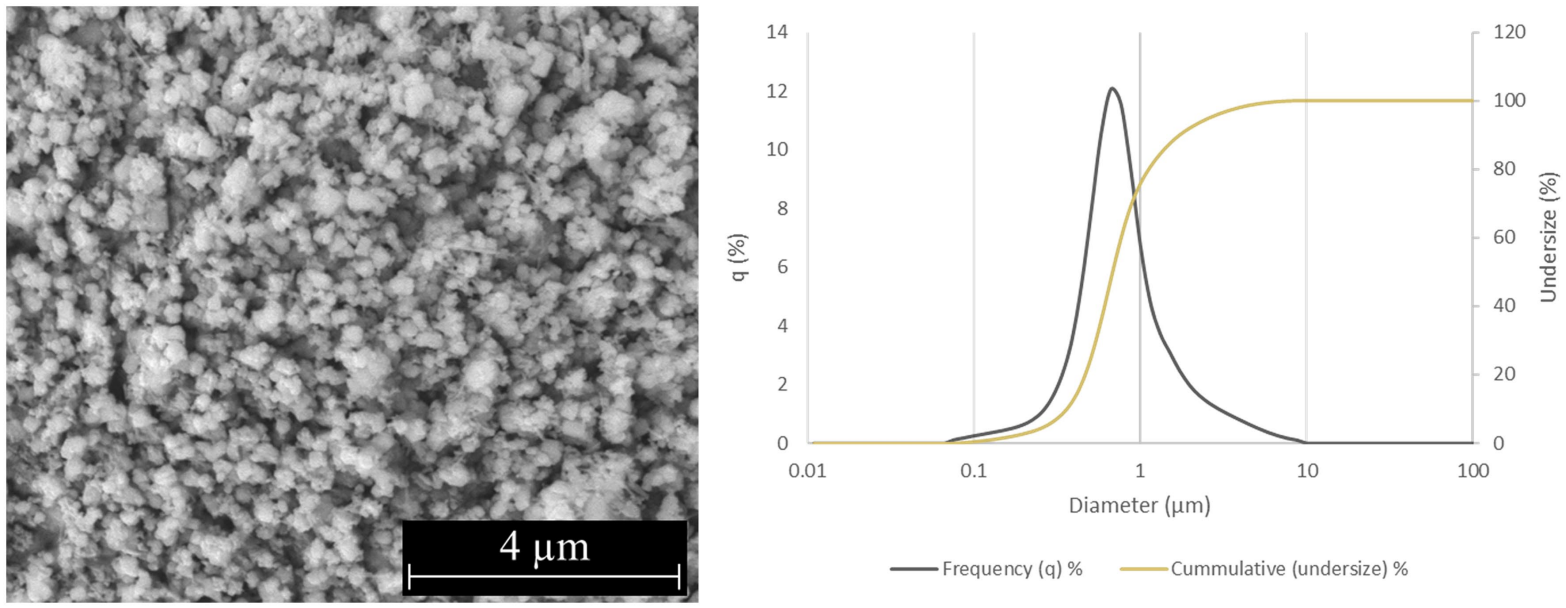
Characterization of α-Si_3_N_4_ (AP^4^) powder. SEM micrograph of AP^4^ at 25,000x and particle size distribution.

**Figure 2:**
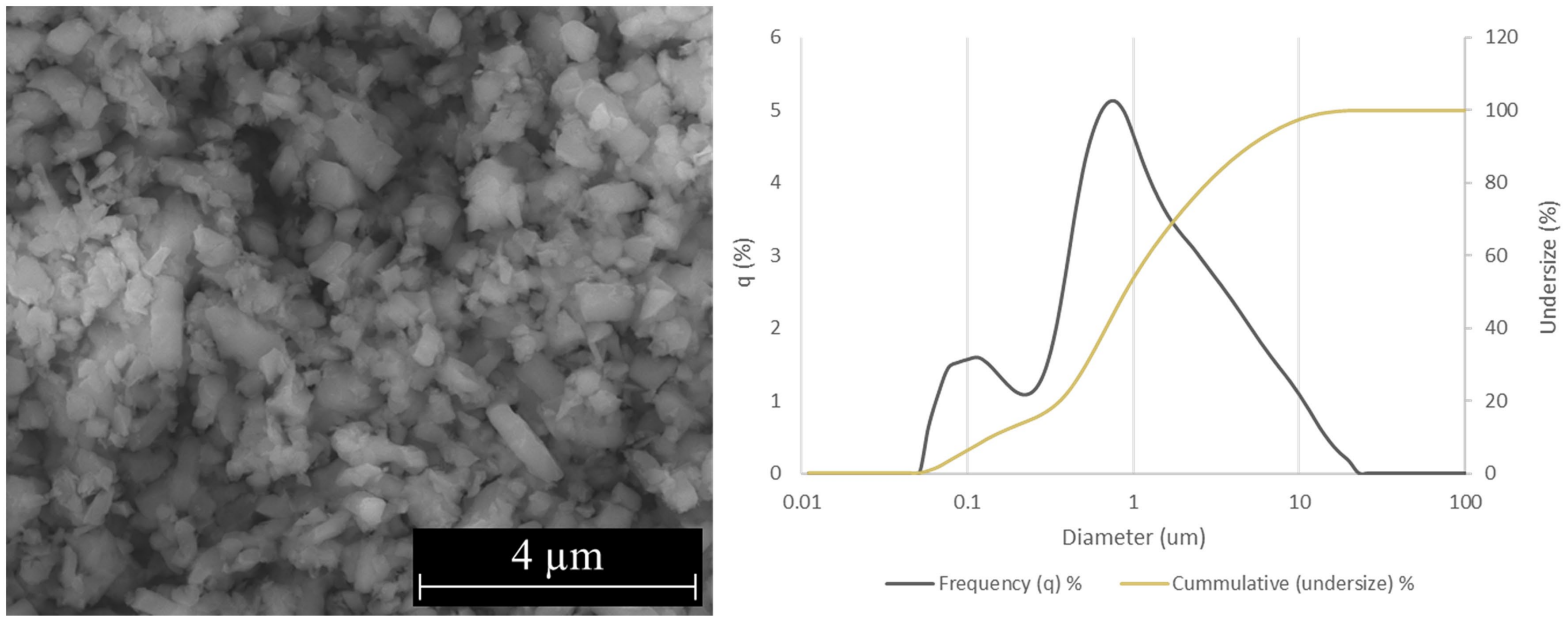
Characterization of β-Si_3_N_4_ (AP^2^) powder. SEM micrograph of AP^2^ at 25,000x and particle size distribution.

Two forms of Si_3_N_4_ powders were used in this study: alpha silicon nitride (α-Si_3_N_4_) and sintered beta silicon nitride (β-Si_3_N_4_) prepared as previously described (Simpson et al., 2023). α-Si_3_N_4_ that possessed a median particle size of 0.6843 μm and a specific surface area of 9.5091 +/− 0.0732 m^2^/g was characterized via scanning electron microscopy (SEM) (Figure 1). The SEM analysis reveals a variety of α-Si3N_4_ particle morphologies. The β-Si_3_N_4_ had a median particle size of 0.8904 μm and a specific surface area of 9.1800 +/− 0.0963 m^2^/g. It was also characterized via SEM (Figure 2). The SEM analysis demonstrated the morphology of sintered Si_3_N_4_ particles, equiaxed grains for α-Si_3_N_4,_ and the telltale elongated, hexagonal cross-section grains for β-Si_3_N_4_.

### 2.2 Silicon nitride reduces SARS-CoV-2 infectious viral titers in a time-dependent manner

**Figure 3:**
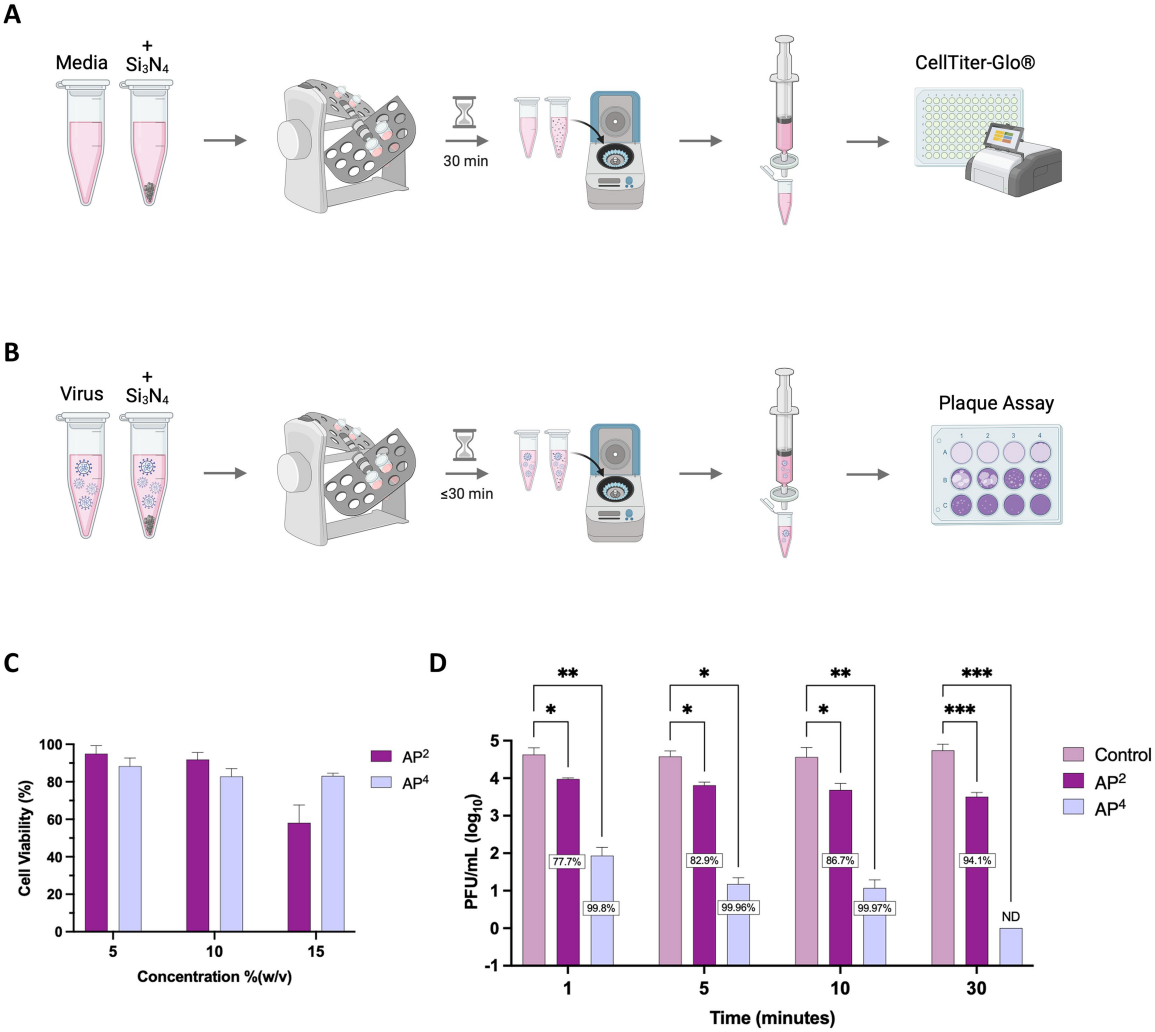
AP^2^ and AP^4^ Si_3_N_4_ powders reduce SARS-CoV-2 in a time-dependent manner. **(A)** Schematic depicting workflow for Si_3_N_4_ powder cell viability. **(B)** Schematic depicting workflow for Si_3_N_4_ powder antiviral testing. **(C)** Clarified supernatants from AP^2^ and AP^4^ at increasing concentrations were incubated with Vero cells for 48 h and assessed for cell viability using CellTiter Glo (*n* = 3). **(D)** Viral titers were measured after incubation of SARS-CoV-2 Lineage A with 15% (w/v) AP^2^ or AP^4^ at increasing exposure time. Control samples are a virus without Si_3_N_4_. Data are mean + SD (*n* = 3, except *n* = 2 for AP^2^ at 1 min and AP^4^ at 5 min). Statistical significance was determined using two-way ANOVA with Dunnett’s multiple comparison test; * *p* < 0.05, ** *p* < 0.01, *** *p* < 0.001, and **** *p* < 0.0001. ND, not detectable. Panel **(A,B)** were created in BioRender. Kehn-hall (2025) https://BioRender.com/c7xzmsl.

Due to the release of reactive nitrogen species when Si_3_N_4_ is exposed to an aqueous environment, we first tested the impact of Si_3_N_4_ on cell viability (Pezzotti, 2018). Vero cells were exposed to clarified supernatants (Figure 3A) from increasing concentrations of α-Si_3_N_4_ (AP^4^) and β-Si_3_N_4_ (AP^2^) for 48 h. Cell viability decreased at the highest powder concentration (15% (w/v)) for AP^4^ and AP^2^ to approximately 80 and 60%, respectively (Figure 3C). Then, the powders were tested at 15% (w/v) for virucidal activity by incubating AP^2^ or AP^4^ with SARS-CoV-2 for 30 min. After incubation, the powders were pelleted by centrifugation, and the infectious virus in the supernatant was assessed via plaque assay (Figure 3B). Both phases of Si_3_N_4_ reduced SARS-CoV-2 (Lineage A) viral titers, as determined via plaque assay in a time-dependent manner (Figure 3D). Exposure of SARS-CoV-2 to AP^2^ resulted in a 0.65–1.23 log_10_ fold change (77.7 to 94.15% inhibition) in viral titers. AP^4^ had a greater impact on viral titer reduction with a 2.7, 3.4, and 3.5 log_10_ fold change (99.8, 99.96, and 99.97% inhibition) at 1, 5, and 10 min, respectively. The greatest decrease in infectious virus occurred when SARS-CoV-2 was exposed to AP^4^ for 30 min, resulting in viral titers falling below detectable limits. Collectively, these results demonstrate that AP^4^ has less cytotoxicity and increased virucidal activity against SARS-CoV-2 (Lineage A) compared to AP^2^.

### 2.3 Reduction in infectious virus is due to silicon nitride activity

**Figure 4:**
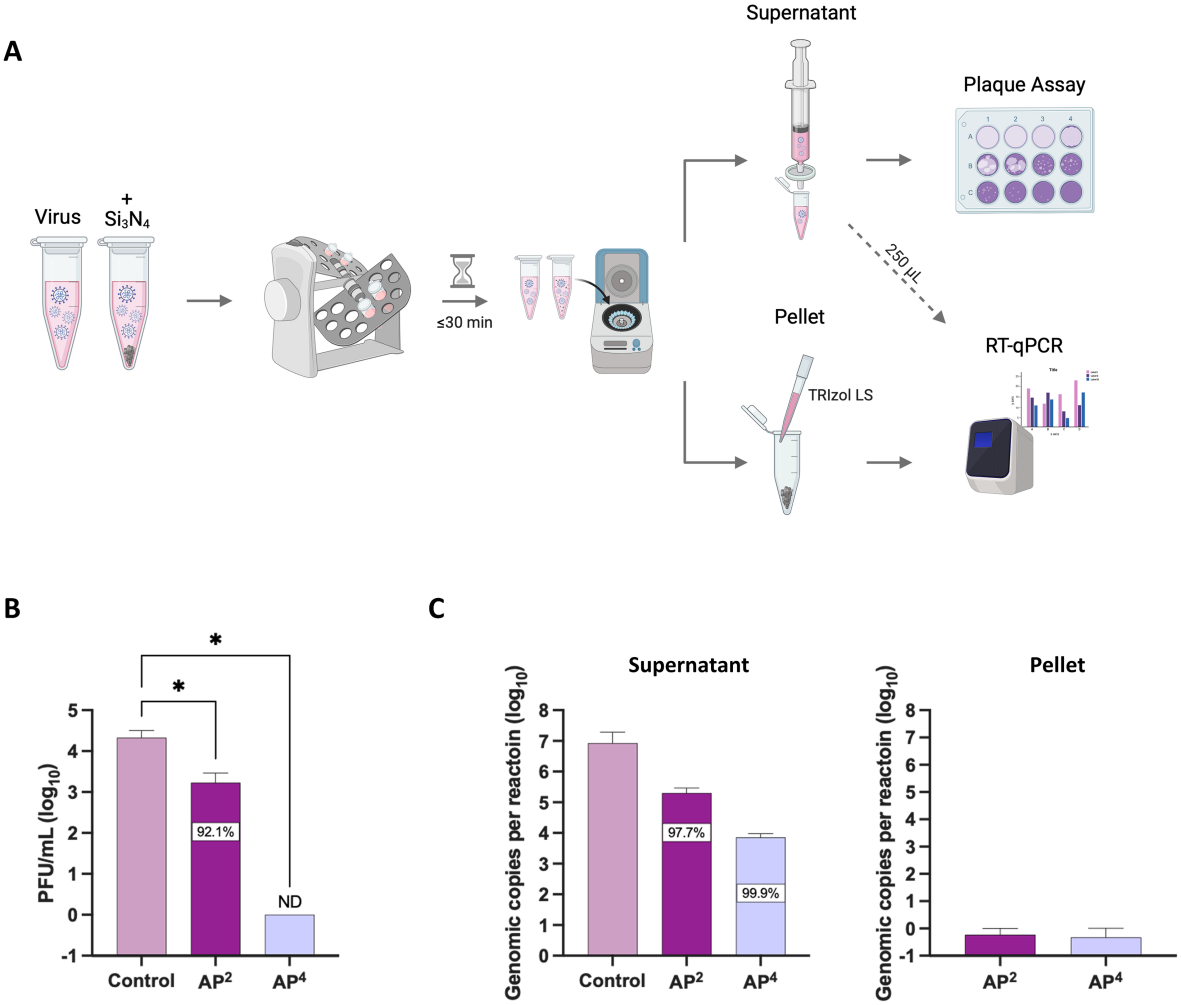
AP^2^ and AP^4^ Si_3_N_4_ powders degrade SARS-CoV-2 viral RNA. **(A)** Workflow of SARS-CoV-2 Lineage A exposed to Si_3_N_4_ powders for concurrent measurement of changes in viral titers and viral RNA. **(B)** SARS-CoV-2 was incubated with powders 15% (w/v) for 30 min to confirm antiviral activity by viral titer reduction. **(C)** Viral RNA levels as determined by RT-qPCR targeting the N gene for SARS-CoV-2 in the clarified supernatant and any residual virus bound to the pellets. Control samples are virus without Si_3_N_4_. Data are mean + SD (*n* = 3). Statistical significance was determined using one-way ANOVA with Dunnett’s multiple comparison test; * *p* < 0.05. ND, not detectable. Panel **(A)** was created in BioRender. Kehn-hall (2025) https://BioRender.com/uw1dvcc.

To further explore the cause for the reduction in viral titers, we sought to test whether the decrease in virus was attributed to the virucidal activity of the Si_3_N_4_ powders or if it was a result of protocol design, where the virus might become bound to the pellets and therefore not being detected in the sample supernatants during plaque assays. SARS-CoV-2 was exposed to Si_3_N_4_ (AP^4^ and AP^2^) at 15% (w/v) for 30 min, with the virus only processed in parallel to serve as a control (Figure 4A). Viral RNA was quantified from clarified supernatants and resuspended Si_3_N_4_ pellets by RT-qPCR. The supernatants were simultaneously used to quantify infectious virus as determined by plaque assay. AP^2^ exposure reduced viral titers by approximately 1.1 log_10_ fold change (92.1% inhibition), consistent with our previous finding, and AP^4^ exposure resulted in zero detectable plaques (Figure 4B). The same pattern of reduction was observed for viral RNA detected in the supernatant by RT-qPCR, with virus exposed to AP^4^ having the largest decrease (99.9% inhibition) in genomic copies per reaction (Figure 4C). Little to no viral RNA (<1 genomic copy for N gene) was detected in the pellets. These findings suggest that the decrease in the infectious virus is due to the antiviral properties of Si_3_N_4_ rather than SARS-CoV-2 being bound to the pellets.

### 2.4 Silicon nitride is a pan-coronavirus virucidal

**Figure 5:**
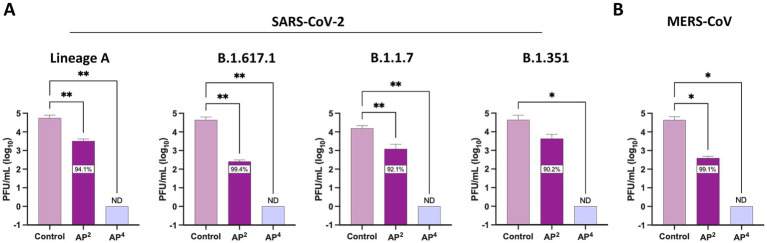
Silicon nitride is a pan-coronavirus virucidal. AP^2^ and AP^4^ 15% (w/v) were incubated for 30 min with **(A)** multiple SARS-CoV-2 lineages and **(B)** MERS-CoV to determine reduction in viral titers. Control samples are a virus without Si_3_N_4_. Data are mean + SD (*n* = 3). Statistical significance was determined using one-way ANOVA with Dunnett’s multiple comparison test; * *p* < 0.05, ** *p* < 0.01. ND, not detectable.

We expanded antiviral testing to include additional SARS-CoV-2 lineages and MERS-CoV to evaluate the pan-coronavirus efficacy of Si_3_N_4_. A reduction in infectious virus was observed across the four SARS-CoV-2 lineages exposed to both AP^2^ and AP^4^. Again, AP^2^ was less effective at reducing viral titers than AP^4^ but had the greatest impact on SARS-CoV-2 B.1.617.1 with a 2.22 log_10_ fold change (99.4% inhibition) (Figure 5A). All viral titers for SARS-CoV-2 lineages fell below detectable limits when exposed to AP^4^ for 30 min. The same trend in viral reduction was observed for MERS-CoV when incubated with Si_3_N_4_, with a 2.04 log_10_ fold change (99.1% inhibition) by AP^2^ and plaques below detectable limits by AP^4^ as compared to the control (Figure 5B). AP^4^ had the greatest pan-coronavirus virucidal activity, outperforming AP^2^.

### 2.5 Fabric infiltrated with Si_3_N_4_ reduces SARS-CoV-2

**Figure 6:**
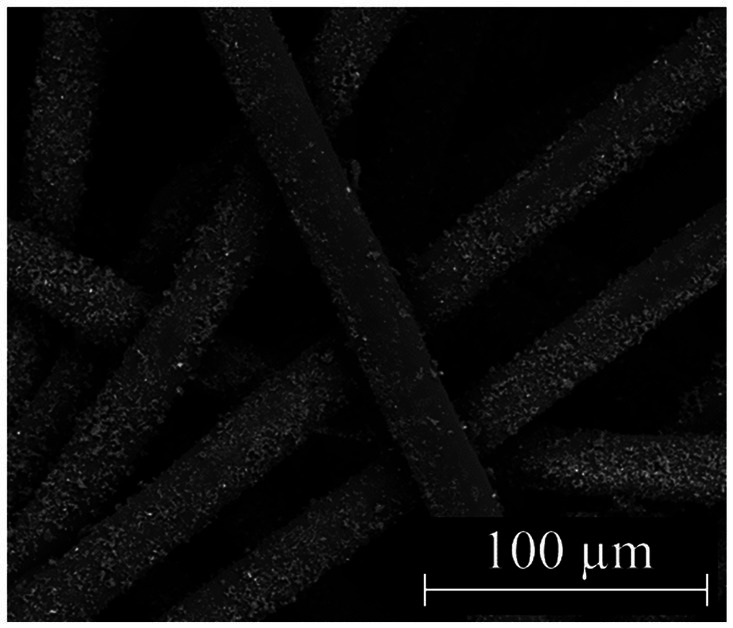
Characterization of fabric infiltrated with Si_3_N_4_. SEM micrograph of dry-infiltrated spunbond polypropylene with α-Si_3_N_4_ at 1,000x.

**Figure 7:**
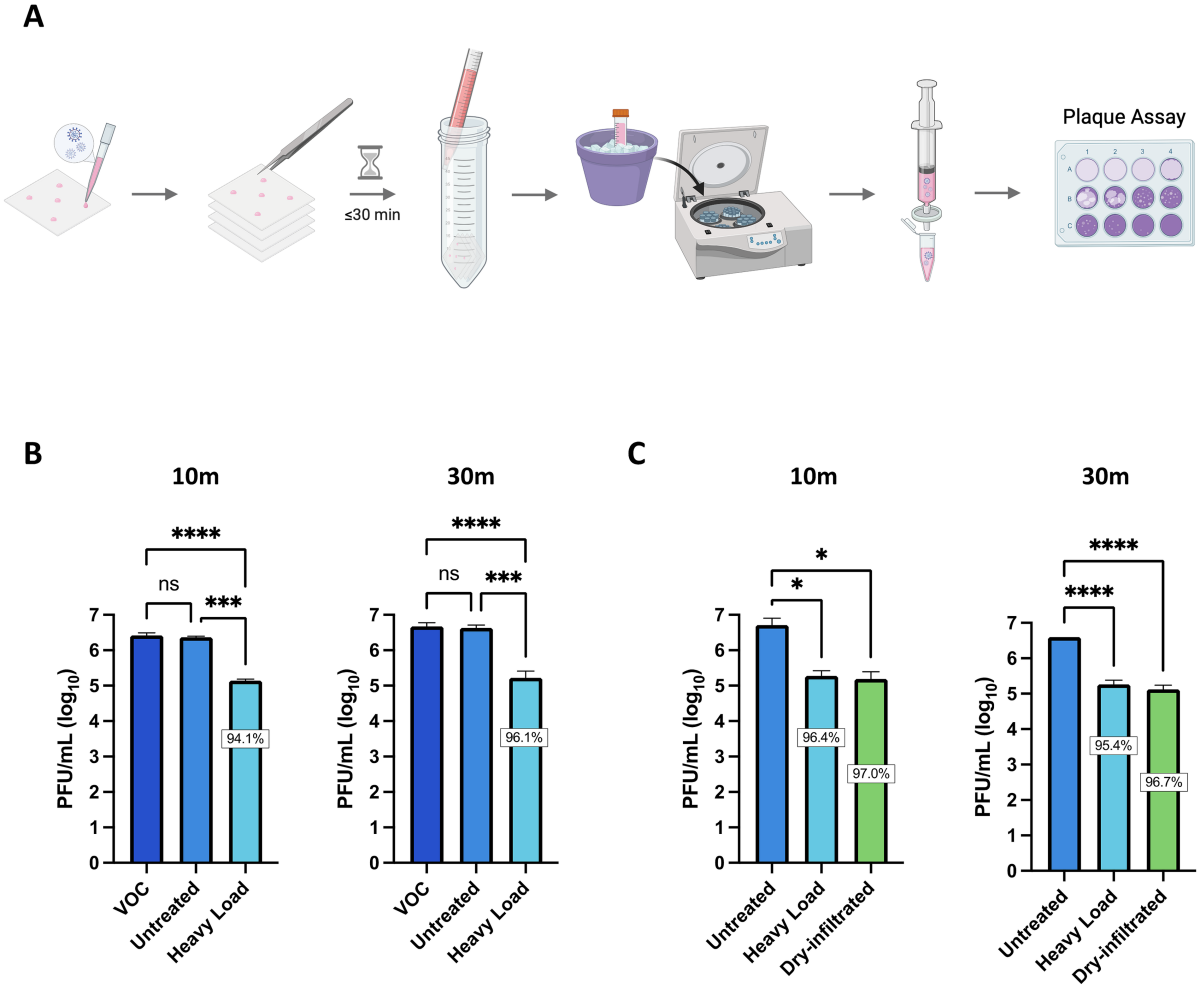
Fabric infiltrated with α-Si_3_N_4_ reduces infectious virus within 10 min of exposure. **(A)** Schematic for testing antiviral activity of α-Si_3_N_4_ embedded fabric against SARS-CoV-2 Lineage A. **(B)** SARS-CoV-2 diluted in media to 2 × 10^7^ PFU/mL was added to untreated control fabric and α-Si_3_N_4_ embedded fabric (heavy load) for 10 (*n* = 3) or 30 min (*n* = 4) and assessed for viral titer reduction. Virus only control (VOC) samples contain only virus and media. **(C)** Redesigned fabric (dry-infiltrated) was compared to heavy load and untreated fabric for viral titers reduction (*n* = 3). Data are mean + SD. Statistical significance was determined using one-way ANOVA with Tukey’s multiple comparison test; ns = not significant, * *p* < 0.05, *** *p* < 0.001, and **** *p* < 0.0001. Panel **(A)** was created in BioRender. Kehn-hall (2025) https://BioRender.com/cwbqc9a.

Our overall scientific objective is to implement Si_3_N_4_-treated personal protective equipment (PPE) as a viral intervention in areas with high-exposure risk (e.g., hospitals). To that end, two types of *α*-Si_3_N_4_-infiltrated fabric were produced and studied. One fabric was infiltrated with AP^4^ (α-Si_3_N_4_) at a heavy load via an aqueous slurry as previously described (Simpson et al., 2023). The second fabric was infiltrated with AP^4^ (α-Si_3_N_4_) using a dry, alternating electric current method as described in the Methods section. SEM analysis of the dry-infiltrated spunbond polypropylene (SBPP) infiltrated with α-Si_3_N_4_-showed fine Si_3_N_4_ particles evenly distributed onto individual polypropylene fibers (Figure 6). Both slurry-infiltrated heavy Si_3_N_4_-loaded fabric and dry-infiltrated fabric were tested for antiviral activity against SARS-CoV-2 (Lineage A) at 10- and 30 min exposure, with testing done in accordance to ISO 18184 test method (Figure 7A). Slurry-infiltrated heavy Si_3_N_4_-loaded fabric was compared to untreated fabric (Figure 7B). To establish a proof of concept for our fabric testing, a virus only control (VOC) (virus in media) was included to determine if equivalent viral titers from the untreated fabric could be obtained. Slurry-infiltrated heavy Si_3_N_4_-load fabric exhibited a 1.28 log_10_ fold change (94.1% inhibition) at 10 min, and a 1.45 log_10_ fold change (96.1% inhibition) at 30 min compared to the untreated fabric. Additionally, no statistically significant differences were observed in viral titers for the untreated fabric and VOC, validating the controls within our experimental design.

We next compared the antiviral activity between the slurry-infiltrated heavy Si_3_N_4_-loaded fabric and the dry-infiltrated fabric. The dry-infiltrated fabric exhibited a 1.48–1.52 log_10_ fold change (96.7–97% inhibition) in viral titers (PFU/mL) when incubated with SARS-CoV-2 for 30 min and 10 min (Figure 7C). The reduction in virus observed for dry-infiltrated fabric was comparable to the positive control, slurry-infiltrated heavy Si_3_N_4_-loaded fabric.

## 3 Discussion

Our studies investigated the antiviral properties of β-Si₃N₄ and α-Si₃N₄ in aqueous suspension against multiple SARS-CoV-2 lineages and MERS-CoV, and a refined version of α-Si₃N₄ embedded in non-woven fabric against SARS-CoV-2 (Lineage A). It has been previously shown that β-Si₃N₄ (AP^2^) can inactivate SARS-CoV-2 and human coronavirus OC43 (HCoV-OC43) (Simpson et al., 2023). Here, we confirmed these data to show AP^2^ can inactivate multiple strains of SARS-CoV-2 and present new findings on its ability to inactivate MERS-CoV. We further expanded testing of Si_3_N_4_ to show that α-Si₃N₄ (AP^4^) powder can inactivate SARS-CoV-2 and MERS-CoV to greater levels than β-Si₃N₄ (AP^2^).

It is hypothesized that the production method for β-Si_3_N_4_ powder is responsible for the observed difference in antiviral activity compared to α-Si₃N₄. The processing of β-Si₃N₄ is believed to accelerate the hydrolysis reaction of the amine functional groups (Si_2_-NH or Si-NH_2_) on the Si_3_N_4_ surface, with the release of NH_3_/NH_4_^+^, contributing to the antiviral mechanism (Pezzotti, 2019; Pezzotti et al., 2021; Pezzotti et al., 2020; Bock et al., 2015). Alternatively, α-Si_3_N_4_ was only subjected to a few moderate cleaning steps to ensure adventitious carbon and other impurities introduced by handling were removed prior to analysis. It is hypothesized that the minimal processing of α-Si_3_N_4_ powder did not impact the surface hydrolysis reaction that drives antiviral activity. Additionally, β-Si₃N₄ is made with sintering aids, i.e., Al_2_O_3_ and Y_2_O_3_, at a concentration of about 10% by mass to facilitate the α-to β-phase transformation during powder sintering (Lange, 2006). The inclusion of these oxides moderately dilutes the concentration of amine functional groups at the surface, thus decreasing the release of NH_3_/NH_4_^+^. The final process step for β-Si_3_N_4_ may also contribute to the diminished antiviral activity compared to α-Si₃N₄. Furthermore, β-Si_3_N_4_ underwent an aggressive aqueous milling cycle at the end of its production process. It is presumed that the powder surfaces were partially hydrolyzed during this milling cycle, further reducing the concentration of available amine sites relative to those available on the high-purity α-Si_3_N_4_. Future studies will include a detailed powder surface characterization to assess these hypothesized differences.

α-Si₃N₄ (AP^4^) powder also demonstrated significant virucidal activity against SARS-CoV-2 when embedded into non-woven fabric, a type of fabric used to make PPE, such as masks. While previous studies have shown the effectiveness of Si_3_N_4_ embedded in non-woven fabric against SARS-CoV-2, issues related to wettability due to uneven distribution of Si_3_N_4_ rendered the fabric in need of further modification (Simpson et al., 2023). Here, we have partially addressed this by designing our experiments using a “sandwich” method in which we stacked four pieces of fabric to meet the ISO 18184 testing fabric weight requirements and increased surface area contact. The refined fabric and optimized study design resulted in greater reduction of SARS-CoV-2 within 10 min of contact with heavy load and dry-infiltrated fabric, resulting in 94.1 and 97% inhibition (Figure 7), compared to 65.8% inhibition at 10 min observed by Simpson et al. (2023). The primary challenge in limiting the spread of SARS-CoV-2 was due to the aerosols generated by infected individuals. Therefore, a limitation of this study is that testing viral droplets while utilizing a “sandwich” method to increase surface area contact may not accurately reflect viral inactivation in these settings. We hypothesized that an enhanced reduction in virus would still occur using our refined Si_3_N_4_ fabric, but future studies are required to determine the time-to-inactivation and percent inhibition of viral titers.

Positive initial antiviral results prompted the assessment of the scalability of both the aqueous slurry process and the dry, alternating electric current method to embed Si_3_N_4_ into fabrics. The aqueous slurry method is scalable and is based on modified conventional continuous fabric processing techniques; however, it would require further development to become suitable for commercial production. The equipment used in the dry, electric current method works effectively with Si_3_N_4_ powder, and only moderate changes would be necessary for larger-scale implementation. The discussion of scalability raises questions about wash resistance and durability, which will be addressed in future studies.

Overall, our results revealed that α-Si_3_N_4_ has a significant impact on pan-coronavirus in multiple media forms (powders and fabric) and are in agreement with previous studies exhibiting the degradation of viral RNA by AP^2^ against multiple SARS-CoV-2 variants (Pezzotti et al., 2022; Pezzotti et al., 2020; Pezzotti et al., 2021). Exposure of viruses to Si_3_N_4_ can damage viral RNA. Specifically, ammonia (NH_3_) and nitric oxide (NO) cause uracil/guanine ring disruption and hydrolysis of RNA phosphodiester bonds (Pezzotti et al., 2021; Pezzotti et al., 2021). RT-qPCR analysis of SARS-CoV-2 revealed that Si_3_N_4_-induced reduction in viral RNA mirrored the decrease in viral titers for AP^2^ (Figure 4). However, AP^4^ had a much more pronounced impact on infectious virus as compared to the reduction of viral RNA, suggesting that mechanisms beyond viral RNA degradation could be impacting infectious virus production upon exposure to AP^4^. Supporting this, Si_3_N_4_ has been shown to induce oxidation of methionine and tyrosine nitration, which can alter protein secondary structure and ultimately affect viral infectivity (Pezzotti et al., 2021).

Viral viability on surfaces relies on evaporation kinetics and surface area, in which mask usage is highly effective in mitigating viral transmission. However, efficacy is dependent on proper fit and mask material (Chatterjee et al., 2021; Ueki et al., 2020). Careful consideration must be taken when choosing mask and fabric type, with respirators and surgical masks most effective at dampening spread, but cloth material leaves the user and bystanders at greater risk of infection (Asadi et al., 2020). Masks can be augmented to have enhanced antiviral properties through the addition of inorganic materials such as zinc, silver, and gold; some of which induce the release of reactive oxygen and nitrogen species from nanoparticles, making these attractive options to incorporate into PPE (Karmacharya et al., 2021; Carvalho and Conte-Junior, 2021). Previous research showed β-Si_3_N_4_ particles in aqueous suspensions inactivate SARS-CoV-2, with antiviral activity similar to copper (Cu), a known virucidal material, and aluminum nitride (AlN), which is hypothesized to also exhibit virucidal properties due to a similar surface hydrolysis-NH_3_/NH_4_^+^ release mechanism as occurs with Si_3_N_4_ (Pezzotti et al., 2021). This study used a similar assay in which supernatants of a 15% by mass dispersion of each material produced a 100% reduction in live virus relative to the control at exposure times of 1 and 15 min. Further, VeroE6 cells exposed to the β-Si_3_N_4_ supernatant did not exhibit a significant toxic response, while limited toxicity was observed following AlN supernatant exposure, and severe toxicity was observed following Cu supernatant exposure. These results suggest a potential for comparable virucidal action without associated toxic effects when β-Si_3_N_4_ is employed instead of AlN or Cu. This finding agrees with the literature that suggests Si_3_N_4_, unlike Cu and AlN, does not exhibit toxicity to mammalian cells (Pezzotti et al., 2020). Potential toxicity of the Si_3_N_4_ powder to humans upon prolonged use has been preliminarily assessed using eukaryotic cell surrogates (such as in this study) and conventional chemical extraction methods. The substrate fabrics used have been through extensive toxicity testing and are currently used in many FDA-approved PPE devices. In-depth toxicity testing on a finished device is planned as part of the next phase of commercialization, but the nontoxic nature of Si_3_N_4_ and its release of reactive nitrogen species make it a promising component to incorporate into non-woven fabrics for PPE.

Although we have shown nontoxic α-Si_3_N_4_ to be advantageous over similar biomaterials in the proposed incorporation into PPE, further research is necessary to determine Si_3_N_4_’s time-to-inhibit upon aerosol exposure, as our study tested SARS-CoV-2 droplets on Si_3_N_4_ embedded fabric. Additionally, other applications for inorganic additives for viral inhibition could be explored, such as incorporating α-Si₃N₄ into HEPA filtration devices. In conclusion, the findings presented here demonstrate the potential of Si₃N₄ as an effective material for broad-spectrum pan-coronavirus inactivation, with promising applications in both suspension and fabric-based solutions for enhancing public health protections.

## 4 Materials and methods

### 4.1 Cell culture

African green monkey kidney epithelial (Vero) cells (ATCC, CCL-81) were maintained in Dulbecco’s Modified Eagle Medium (DMEM) (VWR, 10128-208) supplemented with 10% fetal bovine serum (FBS) (VWR, 97068-085), 1% L-glutamine (VWR, 45000-676), and 1% penicillin/streptomycin (VWR, 45000-652). Vero E6 cells (ATCC, CRL-1586) were maintained in Eagle’s Minimum Essential Medium with L-glutamine (EMEM) (VWR, 10128-608) supplemented with 10% FBS and 1% penicillin/streptomycin. Vero E6-TMPRSS2-T2A-ACE2 (TMPRSS2) cells (BEI Resources, NR-54970) were maintained in DMEM supplemented with 10% FBS, 2% L-glutamine, and 10 μg per mL puromycin (Gibco, A1113803). All cells were maintained at 37 °C in a 5% CO_2_ environment.

### 4.2 Viruses

SARS-CoV-2 isolates hCoV-19/USA-WA1/2020 (Lineage A) (NR-52281), hCoV-19/USA/CA-Stanford-15_S02/2021 (Lineage B.1.617.1; Kappa Variant) (NR-55486), hCoV-19/England/204820464/2020 (Lineage B.1.1.7; Alpha Variant) (NR-54000), hCoV-19/South Africa/KRISP-EC-K005321/2020 (Lineage B.1.351; Beta Variant) (NR-54008), and MERS-CoV (NR-44260) were obtained from BEI Resources. SARS-CoV-2 and MERS-CoV were propagated in Vero E6 cells. SARS-CoV-2 Lineage A was additionally propagated in TMPRSS2 cells when used for fabric antiviral testing as described below. Viral propagation was carried out in the respective media for each cell type with low FBS (2%) and harvested based on the presence of cytopathic effect (CPE) (20–72 hpi). Supernatants were centrifuged (at 21,801 x g for 10 min) to pellet cellular debris and filtered via a 0.2 μm filter (VWR, Cat. #28144-050). The stock virus was titrated according to the plaque assay described below.

### 4.3 Preparation of Si_3_N_4_ powder

AP^4^ and AP^2^ powders were prepared as described previously (Simpson et al., 2023).

### 4.4 Characterization of Si_3_N_4_ powder

#### 4.4.1 Scanning electron microscopy

Samples were imaged using a field emission gun scanning electron microscope (FEG-SEM, Quanta, FEI, Hillsboro, OR, United States). All samples were sputter-coated (108auto, Cressington, Watford, United Kingdom) with a thin (~5 to 30 Å) layer of gold palladium before imaging. Imaging conditions included an accelerating voltage of 10 kV at working distances of 7 to 10 mm and spot sizes of 4 to 4.5 mm.

#### 4.4.2 Particle size analysis

The particle size distribution was measured using a laser scattering particle size analyzer (LA-960A, HORIBA, Kyoto, Japan). The powder sample was dispersed into the measurement medium through a wet flow cell capable of measuring particle size distributions from 10 nm to 5,000 μm using a laser diode light source wavelength of 650 nm for particles with diameters above 500 nm (LED light source wavelength of 405 nm for particles with diameters less than 500 nm).

#### 4.4.3 Specific surface area analysis

The specific surface area was measured using a physisorption analyzer (Gemini V 2365, Micromeritics, Norcross, GA, United States) and calculated in accordance with a multipoint Brunauer–Emmett–Teller (BET) method using dedicated software. The analyzer is capable of measuring specific surface areas ≥ 0.01 m^2^ for a given sample (for a total surface area of ≥ 0.1 m^2^). The samples were prepared by heating (10 °C above ambient to 400 °C) under flowing nitrogen using a degas system (FlowPrep 060 LB, Micromeritics, Norcross, GA, United States). The prepared samples were then analyzed by measuring the volume of gas adsorbed at specific pressures.

### 4.5 Cytotoxicity testing

Vero cells were plated at 2 × 10^4^ cells/well in a 96-well plate (Corning, 3,903). Silicon nitride powder at 5, 10, and 15% (w/v) was added to one mL of complete DMEM supplemented with 10% FBS, 1% L-glutamine, and 1% penicillin/streptomycin. Samples were briefly vortexed prior to 30 min incubation at room temperature with rotation. Samples were then centrifuged at 554 x g at room temperature for 2 min, and the supernatants clarified through filtration with a 0.2 μm filter (VWR, Cat. #28144-050). The clarified supernatants (200 μL/well) were added to Vero cells (2 × 10^4^ cells/well, 96-well plate) (Corning, 3,903) and incubated for 48 h at 37 °C, 5% CO_2_. Untreated cells (complete DMEM) served as the 100% viability control. CellTiter Glo (Promega, Cat #G7570) was used to measure ATP production as a determinant of cell viability.

### 4.6 Powder antiviral testing

SARS-CoV-2 isolates or MERS-CoV were diluted in complete DMEM supplemented with 10% FBS, 1% L-glutamine, and 1% penicillin/streptomycin to a concentration of 2×10^5^ PFU/mL. One mL of virus was added to tubes containing Si_3_N_4_ at 15% (w/v) or without Si_3_N_4_ (virus only) to serve as the control. Samples were briefly vortexed and then incubated at room temperature for up to 30 min with rotation. Samples were then centrifuged at 554 x g at room temperature for 2 min and supernatants clarified through filtration with a 0.2 μm filter (VWR, Cat. #28144-050). Infectious virus was quantified by plaque assay.

### 4.7 Plaque assay

Supernatants for powder antiviral testing were quantified via plaque assay using Vero cells for SARS-CoV-2 isolates (1.5 × 10^5^ cells/well, 12-well plate) and Vero E6 cells for MERS-CoV (2×10^5^ cells/well, 12-well plate). Samples from fabric antiviral testing were quantified using TMPRSS2 cells (2 × 10^5^ cells/well, 12-well plate). Clarified supernatants were serially diluted (1:10), and 200 μL per dilution was added to each well in the 12-well plate. Plates were incubated at 37 °C, 5% CO_2_ for 1 h, rocking every 15 min to ensure adequate coverage of the cells. A 1:1 overlay of 1% agarose (Invitrogen, 16500-500) and 2x EMEM (VWR, Cat # 10128-758) supplemented with 5% FBS, 1% L-glutamine, 2% penicillin/streptomycin, 1% non-essential amino acids (VWR, Cat # 45000-700), and 1% sodium pyruvate (VWR, Cat # 45000-710) were added to the cells (1 mL/well). Plates were incubated at 37 °C, 5% CO_2_ for 48 h or 72 h, for SARS-CoV-2 isolates and MERS-CoV, respectively. After incubation, cells were fixed with 10% formaldehyde and stained with 2% crystal violet in 20% ethanol for counting.

### 4.8 RNA extraction and RT-qPCR

SARS-CoV-2 isolates hCoV-19/USA-WA1/2020 (Lineage A) were exposed to Si_3_N_4_ for 30 min, as written in the powder antiviral testing section. The clarified supernatants were divided for plaque assay, and 250 μL was then transferred to clean microcentrifuge tubes for RNA extraction. Residual media from the Si_3_N_4_ pellets were removed and discarded. Pellets were resuspended in 250 μL complete DMEM.

The pellets and clarified supernatants were then extracted for RNA, in which 750 μL TRIzol LS (Thermo Fisher Scientific, Cat. #10-296-028) was added and the samples vortexed and incubated at room temperature for 5 min. Chloroform (200 μL) (MP Biomedicals, Cat #194002) was added to the samples, vortexed, and incubated at room temperature for 5 min. Samples were briefly vortexed post-incubation and centrifuged at 13,845 x g at room temperature for 10 min. The upper aqueous phase was transferred to a fresh microcentrifuge tube and processed for RNA with the RNeasy Mini Kit (Qiagen, Cat. #74106) using an adapted protocol. RLT buffer (600 μL) was added to the samples and mixed, followed by the addition of absolute ethanol (700 μL), and the samples were mixed. Further sample processing was done in accordance with manufacture’s guidelines (Quick-Start Protocol RNeasy Mini Kit, Part 1). Samples were eluted in 35 μL molecular grade water (VWR, Cat. #95000-094).

Viral RNA was quantified via RT-qPCR using 5 μL of purified RNA with the RNA UltraSense One-Step RT-PCR System (Thermo Fisher Scientific, Cat. #11732927) and a primer-probe set for 2019-nCoV-N1 (IDT, Cat. #10006770), targeting the nucleocapsid (N) gene, on a QuantStudio 3 Real-Time PCR System (Applied Biosystems, Cat. #A28567). Thermal cycling conditions were adapted from the RNA UltraSense One-Step RT-PCR System as follows: 50 °C for 15 min, 95 °C for 2 min, and 40 cycles of 95 °C for 15 s and 55 °C for 30 s. Absolute quantification was determined based on standard curves generated from serial dilutions (1:10) of quantitative synthetic SARS-CoV-2 RNA (BEI, NR-52358) containing fragments from the ORF 1ab, Envelope (E), and Nucleocapsid (N) regions.

### 4.9 Treated textile preparation

Spunbond polypropylene (SBPP) was pre-cleaned and pre-treated as previously described (Simpson et al., 2023). SBPP was infiltrated with α-Si_3_N_4_ and polybutylene succinate (PBS) using a commercial alternating electric current technology (D-Preg, Fibroline, Lyon, France). The α-Si_3_N_4_-PBS powder blend (50 wt.% α-Si_3_N_4_/50 wt.% PBS) was scattered on the SBPP surface, and an alternative electric field was applied to charge the powder, break up the powder agglomerates, and impregnate the non-woven textile. The α-Si_3_N_4_-impregnated SBPP was thermally treated with a heat press at 130 °C at 1 bar for 30 s to melt the PBS binder and allow the α-Si_3_N_4_ particles to adhere or embed into the substrate. SEM characterization was performed on the final treated fabric (Figure 6). Antiviral test swatches of α-Si_3_N_4_-impregnated SBPP were prepared by cutting 25 mm squares and ultrasonically welding three squares together. Four three-layer squares weighing approximately 0.40 ± 0.01 g each were used as one sample for antiviral testing in accordance with the standard ISO 18184 test method.

### 4.10 Textile antiviral testing

SARS-CoV-2 isolate hCoV-19/USA-WA1/2020 (Lineage A) was diluted in complete DMEM to a concentration of 1×10^7^ PFU/mL. Four squares of fabric constituted one sample. Virus was spotted five times onto each fabric square in 10 μL spots, totaling 200 μL of virus per sample. The four pieces of fabric were sandwiched together and placed in 50 mL conical tubes. Virus only (200 μL) control was added to an empty 50 mL conical tube for processing in parallel. Tubes were incubated at room temperature for 30 min. After incubation, 20 mL complete DMEM was added to the fabric. Samples were kept on ice the entire time after the addition of media. Tubes were vortexed 5 times (5 s per time). Ten mL of homogenate was transferred to a 15 mL conical tube. Tubes were centrifuged at 1,256 x g at 4 °C for 2 min and supernatants clarified through filtration with a 0.2 μm filter. Infectious virus was quantified by plaque assay.

### 4.11 Statistical analysis

Samples consisted of three biological replicates unless specified otherwise. Statistical analyzes were performed using GraphPad Prism Software Version 10.4.1. Tests used to determine statistical significance are described within the figure legends. * *p* < 0.05, ** *p* < 0.01, *** *p* < 0.001, and **** *p* < 0.0001.

## Data Availability

The raw data supporting the conclusions of this article will be made available by the authors, without undue reservation.

## References

[ref1] Arts M. P. Wolfs J. F. C. Corbin T. P. (2017). Porous silicon nitride spacers versus PEEK cages for anterior cervical discectomy and fusion: clinical and radiological results of a single-blinded randomized controlled trial. Eur. Spine J. 26, 2372–2379. doi: 10.1007/s00586-017-5079-6, PMID: 28382392

[ref2] Asadi S. Cappa C. D. Barreda S. Wexler A. S. Bouvier N. M. Ristenpart W. D. (2020). Efficacy of masks and face coverings in controlling outward aerosol particle emission from expiratory activities. Sci. Rep. 10:15665. doi: 10.1038/s41598-020-72798-7, PMID: 32973285 PMC7518250

[ref3] Bahl P. Doolan C. De Silva C. Chughtai A. A. Bourouiba L. Macintyre C. R. (2022). Airborne or droplet precautions for health workers treating coronavirus disease 2019? J. Infect. Dis. 225, 1561–1568. doi: 10.1093/infdis/jiaa189, PMID: 32301491 PMC7184471

[ref4] Ball H. T. McEntire B. Bal B. S. (2017). Accelerated cervical fusion of silicon nitride versus PEEK spacers: a comparative clinical study. Journal of. Spine 6:1000396. doi: 10.4172/2165-7939.1000396

[ref5] Bock R. M. Mcentire B. J. Bal B. S. Rahaman M. N. Boffelli M. Pezzotti G. (2015). Surface modulation of silicon nitride ceramics for orthopaedic applications. Acta Biomater. 26, 318–330. doi: 10.1016/j.actbio.2015.08.014, PMID: 26302831

[ref6] Calvert G. C. Huffmon Iii G. V. Rambo W. M. Jr. Smith M. W. McEntire B. J. Bal B. S. (2019). Clinical outcomes for anterior cervical discectomy and fusion with silicon nitride spine cages: a multicenter study. J. Spine Surg. 5, 504–519. doi: 10.21037/jss.2019.11.17, PMID: 32043001 PMC6989924

[ref7] Calvert G. C. Huffmon G. V. B. III Rambo W. M. Jr. Smith M. W. McEntire B. J. Bal B. S. (2020). Clinical outcomes for lumbar fusion using silicon nitride versus other biomaterials. J. Spine Surg. 6, 33–48. doi: 10.21037/jss.2019.12.11, PMID: 32309644 PMC7154368

[ref8] Carvalho A. P. A. Conte-Junior C. A. (2021). Recent advances on nanomaterials to COVID-19 management: a systematic review on antiviral/Virucidal agents and mechanisms of SARS-CoV-2 inhibition/inactivation. Global Chall. 5:2000115. doi: 10.1002/gch2.202000115, PMID: 33786199 PMC7994982

[ref9] Chatterjee S. Murallidharan J. S. Agrawal A. Bhardwaj R. (2021). Why coronavirus survives longer on impermeable than porous surfaces. Phys. Fluids 33:021701. doi: 10.1063/5.0037924, PMID: 33746485 PMC7978145

[ref10] Hardie D. Jack K. H. (1957). Crystal structures of silicon nitride. Nature 180, 332–333. doi: 10.1038/180332a0

[ref11] He Y. Liu W. J. Jia N. Richardson S. Huang C. (2023). Viral respiratory infections in a rapidly changing climate: the need to prepare for the next pandemic. EBioMedicine 93:104593. doi: 10.1016/j.ebiom.2023.104593, PMID: 37169688 PMC10363434

[ref12] Heimann R. B. (2023). Silicon nitride ceramics: structure, synthesis, properties, and biomedical applications. Materials 16:5142. doi: 10.3390/ma16145142, PMID: 37512416 PMC10383158

[ref13] Karmacharya M. Kumar S. Gulenko O. Cho Y.-K. (2021). Advances in facemasks during the COVID-19 pandemic era. ACS Appl. Bio Mater. 4, 3891–3908. doi: 10.1021/acsabm.0c01329, PMID: 35006814

[ref14] Keusch G. T. Amuasi J. H. Anderson D. E. Daszak P. Eckerle I. Field H.. (2022). Pandemic origins and a one health approach to preparedness and prevention: solutions based on SARS-CoV-2 and other RNA viruses. Proc. Natl. Acad. Sci. 119:e2202871119. doi: 10.1073/pnas.2202871119, PMID: 36215506 PMC9586299

[ref15] Lange F. F. (2006). The sophistication of ceramic science through silicon nitride studies. J. Ceram. Soc. Jpn. 114, 873–879. doi: 10.2109/jcersj.114.873, PMID: 28588173

[ref16] McEntire B. J. Maslin G. Bal B. S. (2020). Two-year results of a double-blind multicenter randomized controlled non-inferiority trial of polyetheretherketone (PEEK) versus silicon nitride spinal fusion cages in patients with symptomatic degenerative lumbar disc disorders. J. Spine Surg. 6, 523–540. doi: 10.21037/jss-20-588, PMID: 33102889 PMC7548827

[ref17] Meadows A. J. Stephenson N. Madhav N. K. Oppenheim B. (2023). Historical trends demonstrate a pattern of increasingly frequent and severe spillover events of high-consequence zoonotic viruses. BMJ Glob. Health 8:e012026. doi: 10.1136/bmjgh-2023-012026, PMID: 37918874 PMC10626885

[ref18] Ngandu N. K. Mmotsa T. M. Dassaye R. Thabetha A. Odendaal W. Langdown N.. (2022). Hospital acquired COVID-19 infections amongst patients before the rollout of COVID-19 vaccinations, a scoping review. BMC Infect. Dis. 22:140. doi: 10.1186/s12879-022-07128-5, PMID: 35144556 PMC8830001

[ref19] Pezzotti G. (2018). A spontaneous solid-state NO donor to fight antibiotic resistant bacteria. Mater. Today Chem. 9, 80–90. doi: 10.1016/j.mtchem.2018.05.004

[ref20] Pezzotti G. (2019). Silicon nitride: a bioceramic with a gift. ACS Applied Materials & amp. Interfaces 11, 26619–26636. doi: 10.1021/acsami.9b07997, PMID: 31251018

[ref21] Pezzotti G. Boschetto F. Ohgitani E. Fujita Y. Shin-Ya M. Adachi T.. (2021). Mechanisms of instantaneous inactivation of SARS-CoV-2 by silicon nitride bioceramic. Mater. Today Bio 12:100144. doi: 10.1016/j.mtbio.2021.100144, PMID: 34632359 PMC8485720

[ref22] Pezzotti G. Boschetto F. Ohgitani E. Fujita Y. Zhu W. Marin E.. (2021). Silicon nitride: a potent solid-state bioceramic inactivator of ss RNA viruses. Sci. Rep. 11:2977. doi: 10.1038/s41598-021-82608-3, PMID: 33536558 PMC7858580

[ref23] Pezzotti G. Ohgitani E. Fujita Y. Imamura H. Shin-Ya M. Adachi T.. (2022). Raman fingerprints of the SARS-CoV-2 Delta variant and mechanisms of its instantaneous inactivation by silicon nitride bioceramics. ACS Infect. Dis. 8, 1563–1581. doi: 10.1021/acsinfecdis.2c00200, PMID: 35819780

[ref24] Pezzotti G. Ohgitani E. Shin-Ya M. Adachi T. Marin E. Boschetto F.. (2020). Rapid inactivation of SARS-CoV-2 by silicon nitride, copper, and aluminum nitride. doi: 10.1101/2020.06.19.159970 PMC8485720 34632359

[ref25] Simpson S. McMinn C. Van Mondfrans S. M. Hendry J. Ronayne S. Dewhurst S.. (2023). A novel Antipathogenic agent for nonwoven fabric. Biomed. Mater. Devices 1, 469–482. doi: 10.1007/s44174-022-00001-8, PMID: 40479404 PMC9299416

[ref26] Smith M. W. Romano D. R. McEntire B. J. Bal B. S. (2018). A single center retrospective clinical evaluation of anterior cervical discectomy and fusion comparing allograft spacers to silicon nitride cages. J. Spine Surg. 4, 349–360. doi: 10.21037/jss.2018.06.02, PMID: 30069528 PMC6046334

[ref27] Ueki H. Furusawa Y. Iwatsuki-Horimoto K. Imai M. Kabata H. Nishimura H.. (2020). Effectiveness of face masks in preventing airborne transmission of SARS-CoV-2. mSphere 5:10-1128. doi: 10.1128/mSphere.00637-20, PMID: 33087517 PMC7580955

[ref28] Van Doremalen N. Bushmaker T. Morris D. H. Holbrook M. G. Gamble A. Williamson B. N.. (2020). Aerosol and surface stability of SARS-CoV-2 as compared with SARS-CoV-1. N. Engl. J. Med. 382, 1564–1567. doi: 10.1056/NEJMc2004973, PMID: 32182409 PMC7121658

[ref29] Wang C. M. Pan X. Rühle M. Riley F. L. Mitomo M. (1996). Silicon nitride crystal structure and observations of lattice defects. J. Mater. Sci. 31, 5281–5298. doi: 10.1007/BF01159294

[ref30] Williams B. A. Jones C. H. Welch V. True J. M. (2023). Outlook of pandemic preparedness in a post-COVID-19 world. NPJ Vaccines 8:178. doi: 10.1038/s41541-023-00773-0, PMID: 37985781 PMC10662147

[ref31] Zhu X. Sakka Y. (2008). Textured silicon nitride: processing and anisotropic properties. Sci. Technol. Adv. Mater. 9:033001. doi: 10.1088/1468-6996/9/3/033001, PMID: 27877995 PMC5099652

